# Correction: Reduced protein-coding transcript diversity in severe dengue emphasises the role of alternative splicing

**DOI:** 10.26508/lsa.202402882

**Published:** 2024-06-14

**Authors:** Priyanka Mehta, Chinky Shiu Chen Liu, Sristi Sinha, Ramakant Mohite, Smriti Arora, Partha Chattopadhyay, Sandeep Budhiraja, Bansidhar Tarai, Rajesh Pandey

**Affiliations:** 1 Division of Immunology and Infectious Disease Biology, INtegrative GENomics of HOst-PathogEn (INGEN-HOPE) Laboratory, CSIR-Institute of Genomics and Integrative Biology (CSIR-IGIB), Delhi, India; 2 Academy of Scientific and Innovative Research (AcSIR), Ghaziabad, India; 3 Max Super Speciality Hospital (A Unit of Devki Devi Foundation), Max Healthcare, Delhi, India

## Abstract

Transcriptomic analysis of dengue-infected patients reveals altered immune response pathways, transcript diversity, and splicing efficiency, underscoring potential therapeutic targets for treatment.

Article: Mehta P, Liu CSC, Sinha S, Mohite R, Arora S, Chattopadhyay P, Budhiraja S, Tarai B, Pandey R (2024 Jun 3) Reduced protein-coding transcript diversity in severe dengue emphasises the role of alternative splicing. Life Sci Alliance 7(8): e202402683. doi: 10.26508/lsa.202402683. PMID: 38830771.

**Table 1.** Clinical characteristics of the patient cohort.

Clinical parameters	Mild (n = 45)	Moderate (n = 46)	Severe (n = 21)	*P*-values
Age	27 (18–33)	26 (19–31.75)	35 (18–43)	0.14
Gender F|M	15|30	17|29	4|17	0.34
NS1 antigen	3.22 (0.83–3.60)	3.5 (0.86–3.5)	3.5 (1.9–3.5)	0.75^a^
Total leucocyte count (TLC)	5 (4.6–5.9)	3 (2.5–3.575)	2.7 (2.3–3.1)	**<0.001**
RBC count	4.76 (4.51–4.99)	4.835 (4.58–5.02)	4.78 (4.51–5.58)	0.76
Packed cell, volume	41.2 (40.1–42.8)	42.25 (38.4–44.22)	43.9 (40.4–46.3)	0.18
Platelet count	175 (155–202)	160 (150–165)	125 (112–135)	**<0.001**
Neutrophils	75.9 (66.7–79.5)	63.3 (58.1–68.65)	62.6 (57.3–73)	**<0.001**
Lymphocytes	14.1 (9.4–21.3)	24.5 (20.3–31.975)	26 (15.8–32.7)	**<0.001**
Monocytes	9.8 (8–12)	10 (7.85–11.475)	9 (6.9–11)	0.32
Eosinophils	0.2 (0–0.725)	0.2 (0.1–0.875)	0.5 (0.1–1)	0.32
Basophils	0.5 (0.3–0.6)	0.6 (0.225–0.8)	0.5 (0.3–0.8)	0.69
Hb	13.6 (12.75–14.3)	13.9 (12.52–14.5)	14.1 (13.1–15.3)	0.28
Total protein	7.3 (7–7.6)	6.7 (6.5–7.3)	6.7 (6.05–7.125)	**0.03** ^a^
Albumin	4.4 (4.05–4.5)	4 (3.8–4.15)	4 (3.675–4.1)	**0.04** ^a^
Globulin	3.1 (2.95–3.3)	3 (2.65–3.15)	2.65 (2.3–2.925)	0.06^a^
A.G. ratio	1.4 (1.3–1.55)	1.3 (1.2–1.5)	1.55 (1.375–1.6)	0.19^a^
Bilirubin (total)	0.7 (0.55–0.8)	0.4 (0.3–0.55)	0.75 (0.5–0.9)	**0.02** ^a^
Bilirubin (direct)	0.135 (0.12–0.17)	0.09 (0.07–0.13)	0.17 (0.13–0.23)	**0.02** ^a^
Bilirubin (indirect)	0.56 (0.43–0.615)	0.31 (0.24–0.42)	0.55 (0.39–0.71)	**0.03** ^a^
SGOT- aspartate transaminase (AST)	41 (34.5–76)	59 (44–80.5)	97.5 (74.25–141.5)	**0.03** ^a^
SGPT- alanine transaminase (ALT)	42 (20–95)	41 (25.5–59)	66 (51.75–80.25)	0.13^a^
AST/ALT ratio	0.93 (0.82–1.725)	1.48 (1.13–1.95)	1.705 (1.2–2.17)	0.08^a^
Alkaline phosphatase	67 (60–89)	80 (59.5–100)	82.5 (59.75–124.25)	0.61^a^
GGTP (gamma GT), serum	37 (24.5–46.5)	19 (14–41)	49 (32–82)	0.08^a^

Data represented as median (IQR) or n (%).

a Incomplete data points in either group.

## Supplementary Material

Reviewer comments

