# Supplementary material for: Correction: Reduced protein-coding transcript diversity in severe dengue emphasises the role of alternative splicing
Source: Life Sci Alliance. 2024 Jun 14;7(9):e202402882. doi: 10.26508/lsa.202402882 (PMC11178938; doi:10.26508/lsa.202402882)
Supplement: Reviewer comments [file LSA-2024-02882_review_history.pdf]

Life Science Alliance

Correction: Reduced Protein-Coding Transcript Diversity in Severe Dengue emphasize role of Alternative Splicing

Priyanka Mehta, Chinky Chen Liu, Sristi Sinha, Ramakant Mohite, Smriti Arora, Partha Chattopadhyay, Sandeep Budhiraja, Bansidhar Tarai, and Rajesh Pandey
DOI: https://doi.org/10.26508/lsa.202402882

Corresponding author(s): Rajesh Pandey, Institute of Genomics and Integrative Biology

Review Timeline:

Submission Date:	2024-06-10
Accepted:	2024-06-11

Scientific Editor: Eric Sawey, PhD

Transaction Report:

(Note: With the exception of the correction of typographical or spelling errors that could be a source of ambiguity, letters and reports are not edited. The original formatting of letters and referee reports may not be reflected in this compilation.)

June 11, 2024

RE: Life Science Alliance Manuscript #LSA-2024-02882

Dr. Rajesh Pandey
Institute of Genomics and Integrative Biology
Mall Road, Delhi
Delhi 110007
India

Dear Dr. Pandey,

Thank you for submitting your Correction entitled "Correction: Reduced Protein-Coding Transcript Diversity in Severe Dengue emphasize role of Alternative Splicing".

IMPORTANT: If you will be unreachable at any time, please provide us with the email address of an alternate author. Failure to respond to routine queries may lead to unavoidable delays in publication.

You can contact the journal office with any questions, contact@life-science-alliance.org

Sincerely,

Eric Sawey, PhD
Executive Editor
Life Science Alliance
http://www.lsajournal.org

June 11, 2024

RE: Life Science Alliance Manuscript #LSA-2024-02882

Dr. Rajesh Pandey
Institute of Genomics and Integrative Biology
Mall Road, Delhi
Delhi 110007
India

Dear Dr. Pandey,

Thank you for submitting your Correction entitled "Correction: Reduced Protein-Coding Transcript Diversity in Severe Dengue emphasize role of Alternative Splicing".

IMPORTANT: If you will be unreachable at any time, please provide us with the email address of an alternate author. Failure to respond to routine queries may lead to unavoidable delays in publication.

You can contact the journal office with any questions, contact@life-science-alliance.org

Sincerely,

Eric Sawey, PhD
Executive Editor
Life Science Alliance
http://www.lsajournal.org
